# Lost time undermines return behavior

**DOI:** 10.1093/pnasnexus/pgaf156

**Published:** 2025-06-24

**Authors:** Linda Hagen, Ed O’Brien

**Affiliations:** Department of Marketing, University of Illinois Chicago, 601 S. Morgan St., Chicago, IL 60607, USA; Department of Behavioral Science, University of Chicago Booth School of Business, 5807 S. Woodlawn Ave., Chicago, IL 60637, USA

**Keywords:** return behavior, change over time, happiness, preferences, COVID-19

## Abstract

People commonly experience long gaps of time between getting to do things they love to do. In principle, the longer it has been since people last enjoyed something, the quicker they should jump at the chance to enjoy it again. In practice, five experiments reveal a case of the opposite: The longer since people's last enjoyable experience, the more they *postpone* returning—in part because they demand their return be “extra special” to offset the wait. This effect emerged across many controlled parameters. For example, participants chose to avoid contacting close friends after large vs. small gaps in contact, all else equal—a choice that undermined their immediate happiness. This effect further extended to COVID-19 contexts, regarding people's returns from lengthy shutdowns: Somewhat nonobviously, we found that participants delayed returning to everyday activities *even longer* (as opposed to jumping back at their first sufficiently good chance) if it meant that they could better mark the occasion. Finally, this effect was uniquely attenuated by helping participants reconstrue any chance to return as “extra special.” Together, these findings suggest that time delays create psychological barriers to returning, which people self-impose. People may increasingly *avoid* contacting loved ones, getting back into rewarding hobbies, and so on, the *longer* it has been since last time, promoting vicious cycles of deferment. Motivating people to return to experiences that would enhance their immediate happiness—experiences they still want to have and are now theirs to take—may be surprisingly difficult.

**Significance Statement**

How do people respond to lost time? In particular, how do people make up for missed experiences that matter for their immediate happiness and well-being? Answering this question bears on many consequential issues, from basic theories of motivation (e.g. better understanding how reward value changes over time), to mental health management (e.g. developing psychological strategies for boosting enjoyment), to behavioral prediction (e.g. accurately forecasting rates of economic return from a pandemic shutdown). We document a somewhat nonobvious answer: People delay further rather than jump back in, even when they want to return and when they have good options to return to. Psychological complications undermine decisions to return to happy experiences—at added temporal and hedonic costs in the present.

## Introduction

In his novel *The Plague*, Camus (1) describes the feeling of getting to return to happy experiences after enduring a long temporal gap, as his protagonists felt upon returning from an epidemic quarantine: “For the sensation, confused perhaps but none the less poignant for that, of all those days and weeks and months of life lost to their love made them vaguely feel they were entitled to some compensation; this present hour of joy should run at half the speed of those long hours of waiting” (pp. 239–240).

Indeed, whether by choice or by chance, people often face long gaps of time between getting to do things they love to do—time slips by. Close friends find years have passed since their last contact. Hard workers look up to realize they have not relaxed for months. Faraway family wonder when they last phoned home. After such “long hours of waiting,” what do people do when they finally get the chance to return to happy experiences?

The obvious answer is that long gaps of time may *hasten* people's return behavior: The longer it has been since people's last time enjoying something, the more people may jump at the chance to enjoy it again. Classic economic models of intertemporal choice highlight people's impatience and present bias, such that people jump to enjoy present rewards even when it means forgoing bigger future rewards (2–5). This idea echoes popular theories of motivation that portray delayed gratification as the exception (vs. rule) of goal pursuit (6, 7). Presumably, such effects are amplified as one's last exposure grows more distant, as longer gaps reset hedonic adaptation (8, 9) and foster rosy memories (10) and nostalgia (11–13)—all fueling the pangs of deprivation (14).

However, this choice to return to happy experiences may be surprisingly psychologically complicated. We consider three potential culprits (although this list is surely nonexhaustive):

First, long (vs. short) gaps of time may create perceived uncertainties about one's preferences (e.g. “I used to love chatting with my old friend—but I don't know whether I would still love it now”: 15). As a result, perceived preference uncertainty may lead people to delay their returns for numerous specific underlying reasons, from straightforward value calculations (e.g. people may simply want to avoid doing a now-potentially unwanted activity) to more complex considerations. For example, the thought of returning to a cherished experience, only to realize that one no longer cherishes it, may threaten people's self-consistency motives (16) and desires to retain the original rosy memory (17), leading people to delay returning “just in case.”

Second, even in cases when people explicitly know that they still cherish the experience, long (vs. short) gaps of time may build perceived rust in their abilities to pull it off happily (e.g. “I know I’d still love to chat with my old friend—but I don't know what to say”: 18, 19). That is, people may worry about procedural or practical constraints that they worry have arisen from so much time having passed, leading people to delay returning in order to buy more time to address such constraints (e.g. so people can gain more “practice time” first, before officially returning).

Third, even in cases when people explicitly know that they still cherish the experience, and even when they perceive no rust in their abilities to still pull it off happily, long (vs. short) gaps of time may inflate demanded value, such that people think the occasion needs to be “extra special” to justify and offset the wait (e.g. “I know I’d still love to chat with my old friend and I know what to say—but it needs to be a *big night out”: 20–23)*—akin to Camus’ description from *The Plague* with which we opened the current paper. Any one nondescript present moment to return may strike people as less special than boundless possible future moments to return, leading people to delay for a bigger perceived payoff. From this compensatory view, people may delay not only for reasons that involve wanting to avoid an especially bad return—they may also sometimes delay for reasons that involve holding out for an especially good one.

Throughout our experiments and also in the General discussion, we will return to these various potential mechanisms and discuss our evidence for vs. against them, including discussing exciting avenues for future research to further tease them apart. For now, we note here up front that none of these potential contributors are mutually exclusive, and critically, they each make the same prediction regarding the key basic effect: Somewhat nonobviously, long gaps of time may sometimes slow (not hasten) people's return behavior. The longer it has been since people's last time enjoying something, the more people may *delay* a good opportunity to enjoy it again.

In experiment 1, we exploited a naturalistic context that allowed us to initially explore this research question: People's return behavior from COVID-19 shutdowns.^a^ In Spring 2020, the COVID-19 pandemic caused widespread shutdowns of hedonic activities (e.g. restaurants, social events). As things began reopening in Spring 2021 (24), we wondered: How did the long gap of time since last getting to enjoy these experiences bear on people's returns to them—among people who had safe, available, and attractive opportunities to which they could return?

**Fig. 1.**
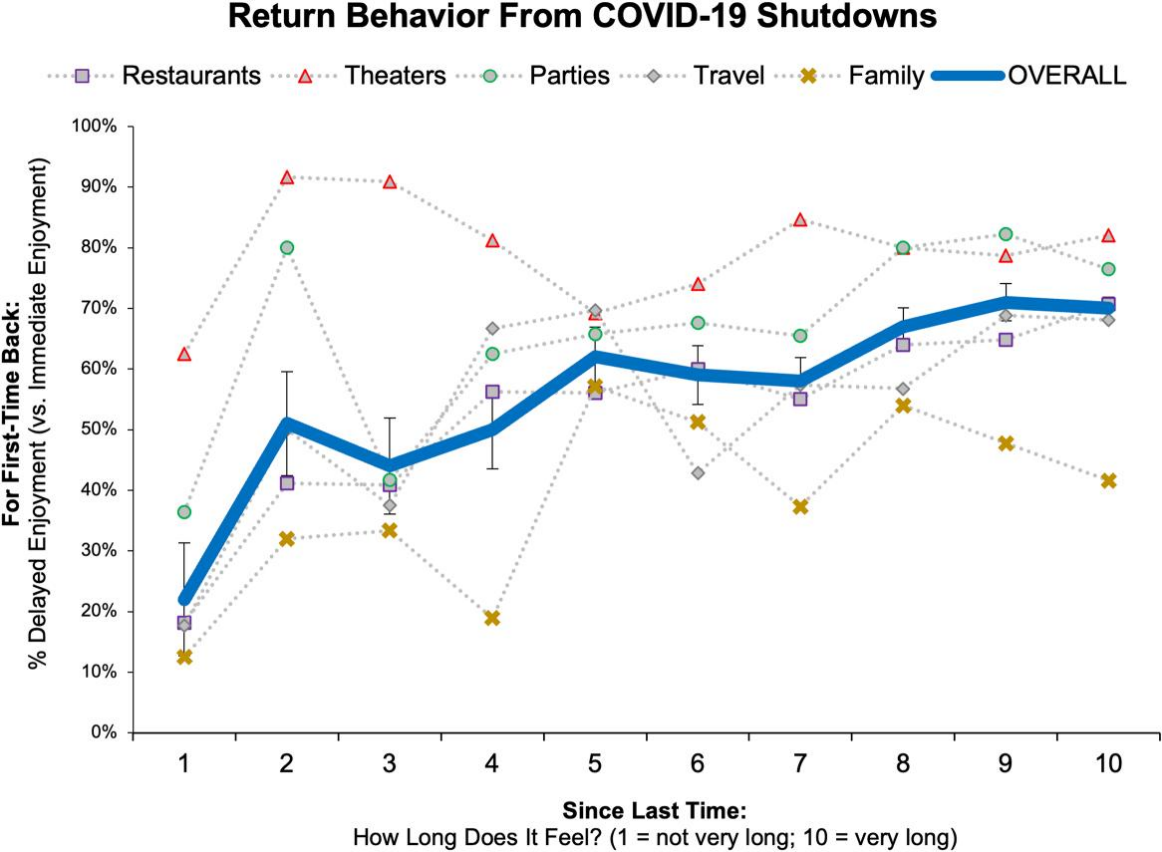
Experiment 1 results: people's return behavior from COVID-19 shutdowns, as a function of how long the gap felt since last time. The figure shows the percentage of responses (*n* = 2,500) at each gap level that entailed delayed enjoyment (over enjoying something that was otherwise immediately available, attractive, and safe to do). “Overall” collapses across all activities (error bars ± 1 SE). We plot the results this way simply for visualize ease. Again, note that the figure plots raw percentages (500 participants for each of five events, within-subjects; the “Overall” line collapses across these events [±1 SE], yielding 2,500 observations). For each event, participants freely rated “How Long” from 1 to 10 (*x*-axis). The resulting sample sizes across these 10 levels thus naturally vary. Across events, they range from: 1 (8–32 *P*'s); 2 (5–25 *P*'s); 3 (11–27 *P*'s); 4 (16–37 *P*'s); 5 (13–41 *P*'s); 6 (27–45 *P*'s); 7 (39–69 *P*'s); 8 (63–75 *P*'s); 9 (44–79 *P*'s); and 10 (123–229 *P*'s). For the “Overall” line, these *n*'s are: 1 (90 obs); 2 (67 obs); 3 (88 obs); 4 (119 obs); 5 (157 obs); 6 (175 obs); 7 (276 obs); 8 (352 obs); 9 (313 obs); and 10 (863 obs).

To find out, we recruited 500 American adults via a national online panel in March 2021 (46% women; 24% non-White; *M*_age_ = 42; 61% college-educated or above; $55,000 median income; 44% married; politics: *M* = 3.54 [1 = extremely liberal, 7 = extremely conservative]; modal location: US states in Eastern Standard Time). Participants reported on their “first-time back” experiences for five activities: dining at restaurants; going to the movies; going to parties; traveling for fun; and visiting family. For each, participants reported: (i) How long the gap of time had felt between their last experience and “first-time back” experience, rated from 1 to 10 such that higher scores reflect a longer felt gap and (ii) what better describes what they did (or will do, if still waiting) for their “first-time back” experience, chosen from one of two options: They returned as soon as they could to enjoy something that was immediately safe, available, and attractive vs. They delayed even longer for a better such option later. By design, note that these choice options circumvent straightforward reasons for delayed returns (rather than immediate returns) that are unrelated to our research question. If participants report a delayed return here, then this cannot simply reflect waiting for safer times or appealing options as their choice comes after such conditions are met. Figure 1 plots participants’ felt gap length as a predictor of their return behavior.

As can be seen, logistic regressions show that longer felt gaps significantly predicted *delayed* (not hastened) returns, with participants waiting even longer for better options later—even though this meant passing up immediately safe, available, and attractive options. This was reflected in a main effect of Gap Length (Wald = 33.73, df = 9, *P* < 0.001), which held across activities (null interaction: Wald = 41.25, df = 36, *P* = 0.252) and return status (null interaction: Wald = 11.03, df = 9, *P* = 0.273). Taking all observations together (*n* = 2,500, across 500 participants each reporting on five return behaviors; see bold blue “Overall” line): Among the 90 responses of “1” to the gap question (meaning participants felt the gap was short), 22% entailed delayed returns rather than immediate returns; yet among the 863 responses of “10” (meaning participants felt the gap was long), 70% entailed delayed returns rather than immediate returns.

Next, experiment 2 moved to controlled laboratory settings to test for the effect in a more common everyday context: contacting close friends. We randomly assigned 200 undergraduates from a large West Coast university in the United States (44% women; 64% non-White; *M*_age_ = 20; 27% freshmen, 44% sophomores, 25% juniors, 4% seniors; 18% international; 72% English first language) to one of two conditions. We instructed Short Gap participants to bring to mind a close friend with whom they have “recently communicated” (we did not specify the length for them; on average, they brought to mind a gap of about 1 week). We instructed Long Gap participants to bring to mind a close friend with whom there has been a “long gap in communication” (we did not specify the length for them; on average, they brought to mind a gap of about 1 year).

The idea here was to test whether people avoid reaching back out to friends merely after a long (vs. short) gap since their last contact, all else equal. To this point, one potential concern we considered was that participants across these conditions may bring to mind qualitatively different friends, which may explain differential choices for reaching out for reasons that are unrelated to the time gap per se (e.g. perhaps Long Gap participants bring to mind less-close friends, which would explain why they may avoid reaching out). To help rule this out, we designed all prompts to ensure participants across conditions brought to mind otherwise similar friends aside from the gap (e.g. participants in both conditions still knew how to contact the friend and felt grateful for the friend, with the gap being caused for no ill reason), and we asked them to rate the friend on relevant dimensions (e.g. participants in both conditions thought that contacting the friend would make both them and the friend very happy). Long Gap and Short Gap participants also rated the friend as equally close at the friendships’ peak. See Supplementary Information for all details and results.

We gave all participants the same choice of what to do in the experiment: They could send a short note of gratitude to their friend (which we chose as a task because past research shows it is a highly rewarding experience: 25) or complete an explicitly dull work task on their own. The choice was real; participants completed whichever task they chose then and there.

Whereas 55% of Short Gap participants chose to write their friend (with the remaining 45% choosing to do the dull work task), 41% of Long Gap participants chose to write their friend (with the remaining 59% choosing to do the dull work task), χ^2^ (1, *n* = 200) = 3.88, *P* = 0.049.^b^

**Fig. 2.**
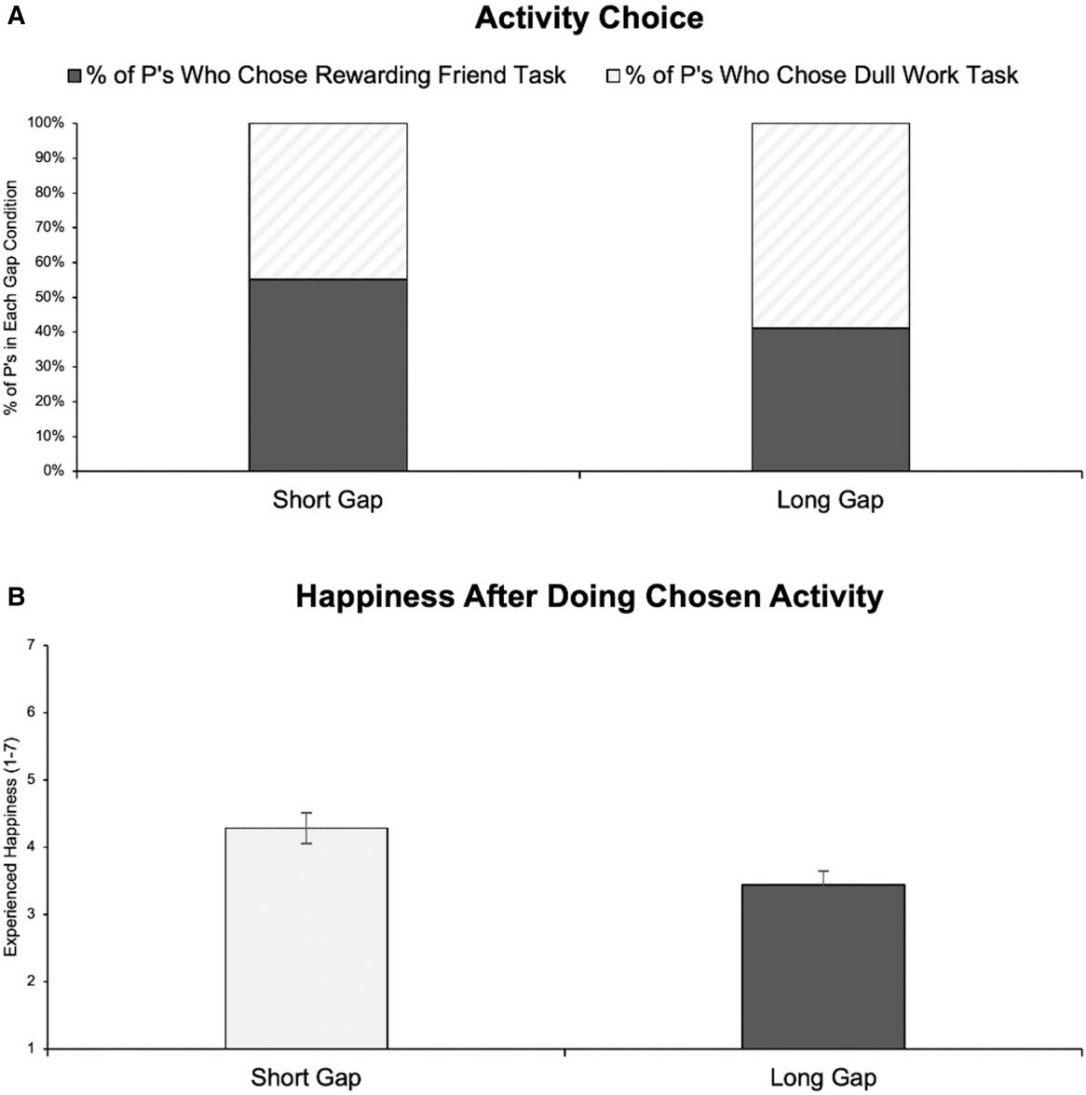
Experiment 2 results: A) choice (raw percentages) and B) happiness (means ± 1 SE).

This choice undermined immediate happiness. Upon finishing their task, participants also completed a six-item (α = 0.98) experienced happiness scale (rated from 1 to 7 such that higher scores reflect greater experienced happiness). Long Gap participants came away less happy (*M* = 3.44, SD = 2.06) than Short Gap participants (*M* = 4.28, SD = 2.25), *t*(198) = 2.75, *P* = 0.006. When rerunning this analysis controlling for task choice via linear regression, the effect size drops in half (from *B* = 0.42, SE = 0.15, *P* = 0.006 to *B* = 0.20, SE = 0.11, *P* = 0.060)—further suggesting it was Long Gap participants’ choice to avoid getting back in touch that spoiled their immediate happiness (beyond, for example, any potential effect of the prompt simply reminding them of the long gap). Figure 2A and B plots these results together.

In experiment 3, we sought to replicate this effect on friend choice with a larger sample while further addressing the “different kind of friend” alternative explanation. We recruited 1,000 American adults (56% women; 34% non-White; *M*_age_ = 37) for a simple texting study (rather than using a “gratitude” task, which may have been uniquely awkward for Long Gap participants in ways we did not fully rule out). We first asked *all* participants to bring to mind *both* kinds of friends—a Short Gap friend who they still text regularly (we did not specify the length for them; on average, they brought to mind a texting gap of about 1 week) and a Long Gap friend who they can still text but, for no ill reason, had not done so in a while (we did not specify the length for them; on average, they brought to mind a texting gap of about 1 year).

We then designed the prompts to be even more explicit (as compared to our between-subjects prompts in experiment 2) about the need for these two friends to be equal to each other in participants’ eyes, in order for the experiment to proceed. We explicitly instructed participants to bring to mind friends who they feel identically about aside from the gap—friends they feel identically close to, and for whom it would be identically pleasant and nonawkward to text with (such that they would feel identically little guilt or nervousness upon texting either). We further clarified that this meant participants needed to bring to mind friends who would elicit equally low social anxiety if they (the participant) were to text them (the friend) and likewise would elicit equally low rejection concerns and equally low concerns about knowing what to say. If participants could not summon two identical friends in all these ways—in effect, if participants could not bring to mind two friends who they viewed as all-else-equal to one another, with the only difference being that participants happened to have a longer incidental gap length since last texting one of the two friends—we instructed participants to exit the experiment at this stage.^c^

We then randomly assigned participants to one of two conditions whereby they chose which task to complete in the experiment. Short Gap participants chose between simply “texting a fun hello” to their Short Gap friend, or completing a “longer and more boring” solo task. Long Gap participants made this same choice except the texting task entailed texting their Long Gap friend.

**Fig. 3.**
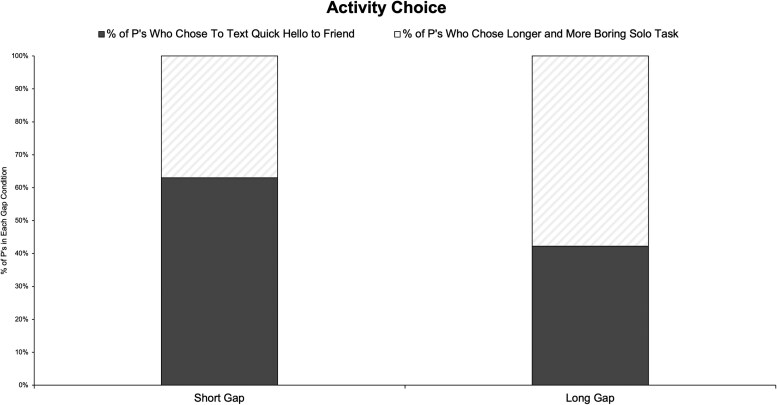
Experiment 3 results: choice (raw percentages).

The effect again replicated: Whereas 63% of Short Gap participants chose to text their friend (with the remaining 37% choosing the longer and more boring solo task), 46% of Long Gap participants chose to text their friend (with the remaining 54% choosing the longer and more boring solo task), χ^2^ (1, *n* = 1,000) = 28.96, *P* < 0.001. Figure 3 plots these results.

Also informative in this experiment, we went beyond the “different kind of friend” issue altogether by measuring participants’ own individual differences in their trait social anxiety (26) and their trait rejection sensitivity (27). Participants’ trait social anxiety did not interact with the effect of gap condition on task choice (null interaction: *B* = −0.03, SE = 0.02, *P* = 0.182), nor did participants’ trait rejection sensitivity (null interaction: *B* = −0.03, SE = 0.03, *P* = 0.326). These findings serve to further validate the fact that we had designed our prompts to avoid participants having such concerns to begin with, thereby suggesting other mechanisms (like the ones we have proposed; we return to mechanisms soon) for why participants avoided reaching out to Long Gap friends beyond being worried about it per se. Put another way: Even people who are generally happy to socialize appear to delay socializing after long (vs. short) gaps.

Next, experiment 4 moved to a further controlled and generalized design whereby we held the stimulus constant while manipulating gap length (thus isolating causality). We randomly assigned 501 American adults (46% women; 23% non-White; *M*_age_ = 41) to one of two conditions. We instructed Short Gap participants to imagine a “short time” had passed (we did not specify the length for them; on average, they brought to mind a gap of about 1 month) since they last did “something they love to do”—simply because life had gotten in the way—and that they now have an opportunity to do it again. We showed Long Gap participants these same prompts except instructed them to imagine that a “long time” had passed (we did not specify the length for them; on average, they brought to mind a gap of about 1 year). As in prior experiments, we did what we could to ensure participants brought to mind otherwise similar experiences aside from the gap (e.g. we asked participants to freely imagine their own activity where such gaps occur for them, and this did not vary by condition; common activities included experiencing nature, traveling, and socializing). All participants reported how much they would prefer to delay the present return opportunity (rated from 1 to 7 such that higher scores reflect stronger delay preferences).

Again, it was Long Gap participants (*M* = 3.44, SD = 1.82), not Short Gap ones (*M* = 2.98, SD = 1.68), who more preferred to delay the return opportunity, *t*(499) = 3.00, *P* = 0.003.

*Why* do long gaps undermine return behavior, all else equal? As discussed, we have considered three (nonexhaustive) culprits: Long awaiters may perceive uncertainty in their preferences, may perceive rust in their abilities, and may inflate demanded value. In cases when the first two possibilities are true (i.e. when people's tastes have actually changed and/or when people are actually rusty after all that time), then people are presumably right to return slowly. The third possibility, however, may be more prone to being illusory, akin to a sunk cost fallacy (28)—it may sometimes feel just as good cashing in on happiness now as it would at a “better” occasion later (29–34), at least in terms of the immediate experience—suggesting people delay happiness needlessly if their goal is to maximize immediate happiness (we further discuss the evidence for—and limits of—this interpretation in the General discussion). We were therefore especially interested in whether this possibility contributes to the effect beyond the former two.

**Fig. 4.**
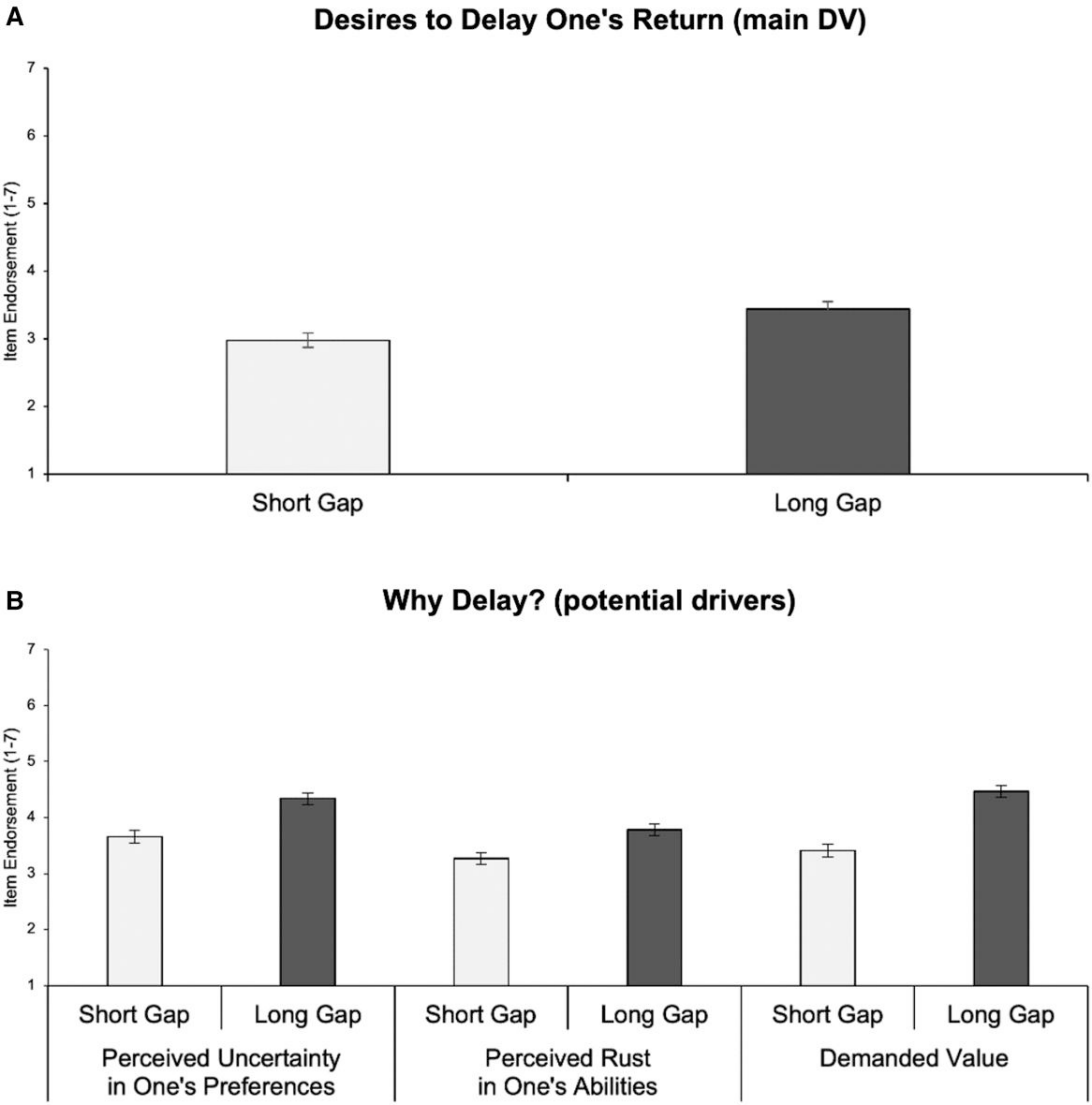
Experiment 4 results: A) desires to delay and B) potential drivers (means ± 1 SE).

Two points of contact suggest it does. First, back in experiment 4, we also asked participants to rate how much the return experience would need to be of exceptionally high value (rated on the same 1–7 scale). Long Gap participants reported stronger endorsement (*M* = 4.47, SD = 1.69) than Short Gap participants did (*M* = 3.41, SD = 1.75), *t*(499) = 6.88, *P* < 0.001—and this difference mediated the effect of the gap manipulation on their desires to delay, *B* = 0.61, SE = 0.10, 95% CI_Boot_ (.43, 0.81). We also asked participants to rate the other two proposed processes: how much the return experience would need to entail certainty in their preferences and entail high abilities (each rated on the same 1–7 scale). Figure 4B plots all these process results together (for reference, Fig. 4A plots the earlier basic effect, i.e. that longer gaps promote more delay).

As can be seen, Long Gap (vs. Short Gap) participants reported stronger endorsement of the other two process items as well, *t*s ≥ 3.33, *P*s ≤ 0.001, which is consistent with our initial theorizing that all three processes may contribute to preferred delay. More relevant here, the statistically strongest effect was for demanded value (interaction between Gap condition and item type: *F*[2, 498] = 7.64, *P* < 0.001). The mediating effect of demanded value remained significant when controlling the other two process items, *B* = 0.18, SE = 0.05, 95% CI_Boot_ (0.10, 0.27).

As a second point of contact, we conducted a fifth and final experiment that utilized a moderation-based approach. Experiment 5 again took advantage of the naturalistic window of COVID-19 return behavior, here to test a strategy for hastening delayed returns. In April 2021, we recruited 599 American adults via a national online panel (52% women; 21% non-White; *M*_age_ = 40; 67% college-educated or above; $65,000 median income; 44% married; politics: *M* = 3.51 [1 = extremely liberal and 7 = extremely conservative]; modal location: US states in Eastern Standard Time) all of whom were still excitedly waiting to have their return experience (as indicated via a prescreen survey; common responses for activity type included travel, concerts, movies, and restaurants). Moreover, all participants reported the gap felt a “10 of 10” long (rated via a 1–10 scale in the prescreen). Thus, all participants assessed a still highly desired and personally relevant activity for which they felt a very long gap. How could they be motivated to return when the time came?

We randomly assigned participants to one of three conditions that assessed different return strategies. Control participants served as a baseline; they proceeded right to the dependent variable, which was the same from experiment 1 (they reported what they will do for their “first-time back” experience: immediate enjoyment vs. delayed enjoyment). Most of these Control participants—64%—preferred delayed enjoyment (with the remaining 36% preferring immediate enjoyment), which replicates experiment 1. For comparison, Present-Special participants also completed this same dependent variable, but we first instructed them to complete a reconstrual task (35) in which they reflected on and wrote about how any occasion to enjoy something can be made to feel like a special one from the right perspective (and indeed, this task led Present-Special participants to view the present as more special than Control participants viewed it, *P* = 0.003; see Supplementary Information for all details and results). Thus, if long awaiters prefer a delay solely due to perceived uncertainty in their preferences or perceived rust in their abilities, then it is not obvious such a task should hasten their return intentions. But it did: Fewer Present-Special participants—51%—preferred delayed enjoyment (with the remaining 49% preferring immediate enjoyment): Control (64%) vs. Present-Special (51%), *B* = −0.59, SE = 0.20, *P* = 0.003.

**Fig. 5.**
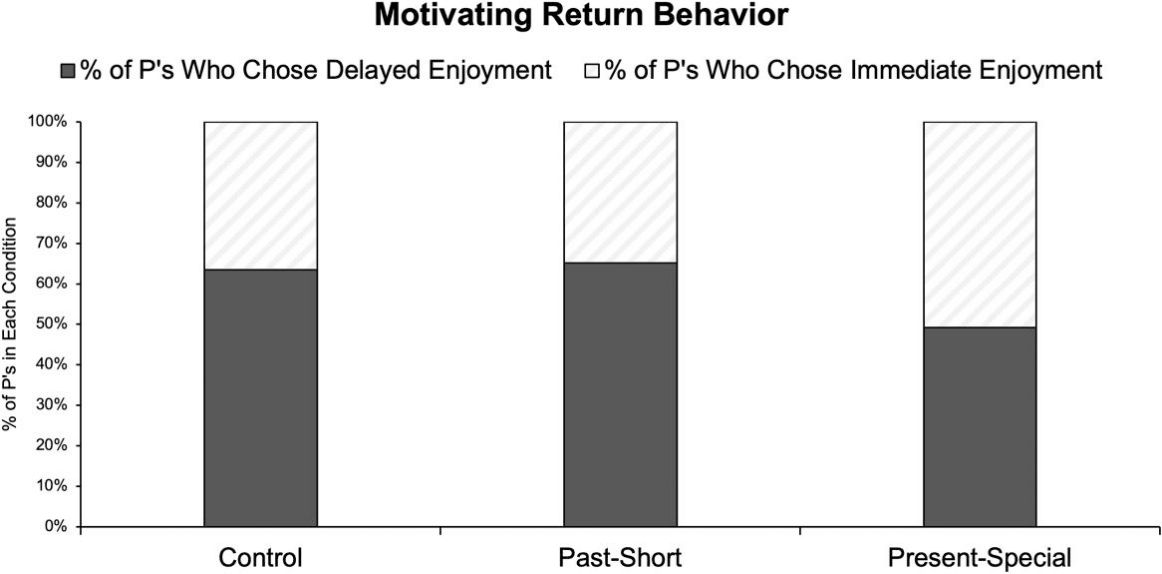
Experiment 5 results: preferences to delay one's return by condition (raw percentages).

Also informative were the participants assigned to the third condition—the Past-Short condition. Past-Short participants completed a different reconstrual task about how any long gap of time can be made to feel like a short one from the right perspective (and indeed, this task led Past-Short participants to view the pandemic gap as shorter than Control participants viewed it, *P* < 0.001; see Supplementary Information for all details and results)—yet despite this, Past-Short participants *did not* hasten their return intentions relative to Control participants (66% preferred delayed enjoyment, with the remaining 34% preferring immediate enjoyment): Control (64%) vs. Past-Short (66%), *B* = 0.07, SE = 0.21, *P* = 0.732. As seen in our preregistration, we initially thought this condition too may generally hasten returns compared with Control participants—but the null result here makes sense from the perspective of isolating different specific mechanisms. For example, this null result is further consistent with the idea that long awaiters delay the return because they demand “the right time” to mark the moment. Shortening the perceived gap on its own proved insufficient to hasten return intentions—presumably because this does nothing to boost people's perception that “now” is the right time (it simply makes “now” feel closer). We thus interpret these results as further highlighting the role of the “demanded value” mechanism. Figure 5 plots all these results together.

## General discussion

The dictionary defines “to make up for lost time” as “to do something *faster* or *more often* to compensate for not having done it quickly or often enough before” (36). This definition may fit better in principle than in practice. The current research reveals a case of long awaiters running into psychological complications that lead them to return to happy experiences more *slowly*—despite wanting to return in principle, and despite having good options to return to. We further found that one reason for long awaiters’ delay behavior is self-imposed: People seek to make up for lost time not with speed or frequency per se, but with *high value* (“After so much time, the return needs to be *really* great!”)—at added temporal and hedonic costs in the present.

### Insights and implications

These findings offer a novel perspective on understanding delay behavior. For example, theories of procrastination often assume procrastinators focus on the *negatives* of a task and thus delay to minimize pain (37–39). However, the current research suggests people who look like negative procrastinators on the surface may sometimes be focused on the *positives* of a task and thus delay in the hope of attaining a more pleasurable payoff. Relatedly, theories of savoring often assume people who postpone desirable experiences must do so because they derive some utility from the anticipation (or else why postpone it?: 40–42). However, the current research suggests postponing desirable experiences may sometimes reflect an underlying *wavering* more than an underlying savoring; people may postpone because a mundane present feels like a wasted mark of the long built-up moment. Consider the classic finding that people are willing to pay more for a hypothetical “kiss from a movie star” if it occurs a few days from now vs. if it occurs right now (41)—which is assumed to be a positive, such that people must do this because they derive pleasure from the anticipation. The current research suggests this assumption may be incomplete; people may sometimes delay because “right now” feels insufficiently special, and so they may spend those few extra days not in pleasurable anticipation but in anxious preparation.

That people appear to treat time and value as compensatory resources more generally suggests they may be less present-biased than assumed. Somewhat nonobviously, people may sometimes be *more* patient when desires are *high*, as they care more to not spoil the experience.

Ours is not the first research to document people eschewing present enjoyment (43, 44). Most relevant here is the finding that people procrastinate enjoyable experiences because they discount future feasibility costs (39). In essence, people think they will have more free time in the future than they have today (45) and thus save discretionary fun for this seemingly freer future—often to their detriment (e.g. people are less likely to ever visit local landmarks when they think they have much vs. little time left to do so; people are less likely to ever redeem gift cards with far vs. close expiration dates). The current research builds on this work by suggesting a broader reason for why people seem to care so much about future feasibility, beyond having more capacity to execute the activity: It also frees them up to make the most of it and maximize enjoyment. This suggests people should especially procrastinate enjoyment when they have such specialness goals, which indeed is what we find here in the context of returning from long gaps.

### Do these findings really reflect wavering rather than savoring?

Readers may wonder whether the current findings really reflect wavering rather than savoring; perhaps, our participants chose to delay because this indeed allowed them to better enjoy today, by way of savoring the anticipation of cashing in at a more befitting future time.

We hope the current findings offer a springboard for future research to further decouple these interpretations. However, there is reason to believe the current findings may at least hint at a wavering component. First, the effect replicated across a wide variety of domains that naturally vary on their relevance to pure savoring. Second, one might predict that a pure savoring account should produce a null effect, such that Short Gap participants delay at the same high rate as Long Gap participants (if people are uniformly motivated by savoring alone), but this was not what we found. Third, recall in experiment 2 that participants’ immediate happiness was reduced by their choice to delay, while a pure savoring account should predict immediate happiness to increase. Fourth, lending novel empirical support here: Back in experiment 3, we had also asked those Long Gap participants who chose the longer and more boring solo task *why* they did not simply choose to text their friend. They reported what better described their rationale, chosen from one of two options: because the wait would be enjoyable and entail savoring the anticipation vs. because they wanted to wait for a “right time” later, despite the wait being unenjoyable. Indeed, 92% of them chose the latter, such that they made this choice despite it being immediately unenjoyable (with only 8%—the remainder—delaying due to savoring), χ^2^ (1, *n* = 268) = 190.58, *P* < 0.001.

### Further insights into specific underlying mechanisms?

As we discuss in the Introduction, the basic effect we observed here—such that long (vs. short) gaps promote delayed returns—is surely multiply determined. More research is needed for further documenting such mechanisms and comparing and contrasting their effects.

For example, readers may still wonder if the “friend” experiments (experiments 2 and 3) operate via unique mechanisms such as feelings of embarrassment for not reaching out sooner, fears of social rejection, or fears about finding common topics after so much time has passed. As we had discussed throughout those experiments, we worked in many ways to control for and rule out such differences—but more research is needed here. For example, it is presumably harder for people to make a social (vs. nonsocial) experience special “on their own,” since the dynamics of the experience also depend on the other person. Likewise, friends can change independently, meaning that people's desires to return to social (vs. nonsocial) experiences may be more likely to truly abate over time—providing a straightforward reason for people's hesitance to return in this domain (i.e. one's friend is no longer one's friend, despite no change in oneself). This fact suggests there must be unique mechanisms that explain people's social (vs. nonsocial) returns, in addition to the evidence for some shared mechanisms as well that we have documented here.

More broadly, taking all domains together, more research should further unpack specific underlying mechanisms. For example, consider again the aforementioned finding that people sometimes delay returning to happy experiences due to strategic memory protection (e.g. people may freely choose to avoid a special spot from their past lest their return is not so special and so dilutes the rosy memory of the original experience: 17). We had grouped this specific possibility under the general mechanism category of perceived preference uncertainty. Although we found some evidence that specialness concerns indeed go beyond perceived preference uncertainty, our experiments may not have fully captured the conditions that specifically trigger strategic memory protection. For example, long awaiters may delay their returns to “getting to travel anywhere in general” (akin to many of the decision tasks we studied) due to specialness concerns, but delay their returns specifically to their single favorite destination due to strategic memory protection.

### Do these findings reflect a “happiness error,” all told?

Another question entails the ultimate take-home message of these findings. Theoretically, the effect reveals a case that counters traditional models of hyperbolic discounting; practically, the effect reveals a case in which people make choices that harm their immediate well-being. But all told, are people “right”/rational or “wrong”/irrational to delay their returns? Answering this question requires one to account for the fuller time-course of utility. While delayed returns cause hits to people's immediate happiness, perhaps these hits are offset by even greater payoffs down the line (e.g. perhaps the experience truly is enhanced upon cashing it at that “right time” later, or otherwise, the delay leaves people with less regret or a more pleasurable memory or story). If people have the goal to maximize their immediate happiness, then the effect indeed reflects a “happiness error”—but it may not be so erroneous if people delay due to other goals like these.

Future research could fruitfully tease apart these ideas further. However, we note here that such delays may indeed at least sometimes be suboptimal. As we discussed earlier, people may risk waiting for the “right time” in vain, as it may not always enhance their eventually experienced enjoyment as much as they think (28–34)—and it may never come in the first place. Seemingly better occasions may perennially lurk in the future (46–48), creating a vicious cycle: A long gap raises the bar for one's return and thus promotes delay—which further lengthens the gap—which further promotes delay—and so on, ad infinitum, with people never enjoying today.

### Other directions for future research

One important limitation of the current research is that, although we assessed real return behavior in experiments 2 and 3 (as well as in experiment 1's “already back” data), we otherwise assessed predicted or intended returns. Future research should further assess real return behavior.

Future research should also further unpack other kinds of activities and activity features. Consider differences in the typical frequency of activities. Echoing our “different kind of friend” explanation that we discussed in experiments 2 and 3, perhaps our Long Gap participants brought to mind rare activities (after all, these were things they had not done for a while), but our Short Gap participants brought to mind common activities (after all, these were things they did recently). If so, then perhaps we simply compared apples to oranges: Long Gap participants may have been more open to further delays not because of the long gap, but because they focused on things they have not done for a long time *and* that they do not expect to do for a long time still. Across our experiments, we worked to control this issue in many ways, including efforts to hold constant the activity itself, but future research should keep working to hold all else equal aside from the gap.

Even when comparing apples to apples, however, readers may still wonder how activity frequency bears on our effect. By design, note that we focused on everyday enjoyable activities to which people might readily return. But not all activities fit this bill. Consider rarely repeated activities where the perception or norm may be for people to return at a low frequency. Different effects could emerge *within* such cases, but still for the same specialness rationale. For example, if people expect to visit a museum just once a year, then short gaps may promote delayed returns as much as long gaps do (e.g. “I just went to the museum yesterday; it’d better *really* be worth it to go again today!”). This idea is partially captured by our COVID-19 return data from experiment 1. As shown in Figure 1, note that one of the five domains showed a nonsignificant effect: returning to movie theaters (*P* = 0.704; all other domains, *P*s ≤ 0.002; see Supplementary Information for all details and results). That is, participants who felt a shorter gap since their last theater experience preferred to delay at a similarly high rate as participants who felt a longer gap. Assuming it is less normative for people to revisit the theater in quick succession (as compared to those other domains like re-encountering the same friends and family), such a result supports this idea. The delay effect may be less pronounced within low-frequency (vs. within high-frequency) activities.

In addition to exploring individual differences across activity type, future research could explore individual and cultural differences across participant type. An especially relevant one pertains to differences between “maximizers” (who generally prefer to select the best possible option, even if it comes at other costs like a higher price or more search effort) vs. “satisficers” (who generally prefer suboptimal but good-enough options that avoid these costs: 49). Readers may wonder whether our effects are simply restating this well-established individual difference: If maximizers are those who are more likely to wait for a bigger payoff, then perhaps our designs inadvertently selected on maximizers only (e.g. the people who can report a long gap are the same people who are also maximizers). We suspect this is unlikely, given that we found that effect via randomly assigned gap lengths (e.g. experiment 4) and that all experiments still found a relative difference between short gaps and long gaps (whereas maximizers should presumably also seek to maximize their experiences after short gaps as well). Thus, we believe our findings suggest the following novel insights on this front: (i) One input into the origins of maximization dispositions may be long gaps (e.g. perhaps the people who become maximizers are those who face long gaps between experiences); (ii) maximizers should show the effect to a greater degree than satisficers; but (iii) satisficers should also show the effect too, simply to a lesser degree.

Finally, these dynamics may more generally extend to other consequential settings like returning to the dating scene (e.g. the longer people go without a date, the *pickier* they may get in acting on good return options—and so act on none). Motivating people to return to things that matter for their immediate happiness and well-being—things they want to have and are theirs to take—may be surprisingly difficult. Long built-up expectations may undermine their realization. For example, regarding COVID-19 return behavior, one headline from 2021 pronounced “42% of People Plan to Throw the “Biggest Party of Their Lives’ When The Pandemic is Officially Over” (50)—terrific in principle, but perhaps stressfully unattainable in practice. Our findings may explain the surprising difficulty of curbing postpandemic demand, despite raised interest rates and other economic sanctions (51). Reconstrual strategies (like the one we verified in experiment 5) may be needed to help people less dramatically and more happily jump back in.

## Materials and methods

All experiments (approved under University of Chicago IRB19-1747; we obtained informed consent from all participants) included various checks (e.g. manipulation, attention, honesty checks; probes for issues/confusions). No fewer than 90% of participants passed each. Results hold when excluding participants otherwise. See Supplementary Information for details.

### Experiment 1

We requested and yielded 500 “Cloud Approved” participants from Cloud Research for $1.00. The experiment followed a five-single-factor design (Event, within-subjects: Restaurants, Theaters, Parties, Travel, Family). We also measured participants’ natural perceptions of Gap Length, which served as our key predictor.

We launched the experiment on Saturday, 2021 March 20. To begin, all participants were informed the experiment was about their own personal “first-time back” experiences—“finally getting to jump back into the fun things you normally do” as “life is now slowly but surely reopening and returning to normal after COVID-19 shutdowns.” The experiment was designed within-subjects, with all participants reporting on their first-time back experiences across five different events, evaluated one by one in random order: “Going out to restaurants for on-premises dining”; “Going out to the movies”; “Going out to indoor parties/larger social gatherings”; “Traveling for fun”; and “Visiting secondary family (i.e. who you don't live with).”

For each event, participants were first asked whether they had already had their first-time back experience, or if they were still waiting (forced-choice, randomly ordered: *already had* vs. *not yet had*). We then asked all participants the same two key dependent variables, shown in random order. Among participants who indicated “already had,” we asked (for each event): “When you had your first-time back experience: How long had it felt? That is, how long did that gap feel between your last previous experience and your first-time back experience? (whenever that occurred),” and they responded from 1 (*actually, not very long*) to 10 (*actually, very long*). We also asked: “For this first-time back experience, what better describes what you did?” They chose from 2 options (forced-choice, randomly ordered): “*I went back out as soon as I could. I wasn't picky; there were good-enough options available, so I excitedly took one*” vs. “*I didn't go back out as soon as I could. I was picky; there were good-enough options available, but I purposefully passed them up, and delayed/postponed until it felt extra special.*” Likewise, we asked participants who indicated “not yet had”: “When you have your first-time back experience: How long will it have felt? That is: How long will this gap have felt between your last previous experience and your first-time back experience? (whenever that occurs),” and, “For this first-time back experience, what better describes what you think you’ll do?,” rated on the same scales.

We also measured other variables simply for interest (e.g. when participants’ return took place; feelings of safety; local status of re-openings). See Supplementary Information for details.

### Experiment 2

We requested and yielded 200 subject pool participants who completed the experiment in individual laboratory sessions for partial course credit. The experiment followed a two-single-factor design (Gap Length, between-subjects: Short, Long).

To begin, all participants were instructed to bring to mind a “close friend” who they “must know how to contact in some way (e.g. you can easily pull up their email/cell/social media/etc.).” Short Gap participants then read: “Please think of a close friend who you have very recently communicated with. Specifically: This must be someone who you feel close to and feel grateful to know, and it's also been very recent since the last time you two communicated.” Long Gap participants then read: “Please think of a close friend who you have not communicated with for a very long time. Specifically: This must be someone who you feel close to and feel grateful to know, but for whatever reason, there's now been a very long gap since the last time you two communicated.” All participants then typed the initials of this person, which we piped into all prompts. They also reported how long it had been since their last contact (open-ended text box).

To further confirm participants brought to mind otherwise similar friends aside from the gap, we measured other variables (e.g. felt closeness; reasons for the gap) and conducted other analyses (e.g. text analysis of the gratitude letters). We also conducted an additional experiment whereby *n* = 100 new Long Gap participants made additional such ratings (e.g. how easy it was to generate the friend; if reaching out would make them and the friend happy; reasons for the gap and if it was due to sour endings or changing tastes). See Supplementary Information for details.

For our main dependent measures, participants were informed they would finish the experiment by completing a task of their choice (with the choices randomly ordered). One was writing a gratitude letter: “All within a strict 5-minute timer: We’ll ask you to write and send a short note of gratitude to [initials], simply expressing how much they mean to you.” The other was completing an explicitly dull transcription task: “All within a strict 5-minute timer: We’ll ask you to start and finish a dull transcription task requiring you to accurately copy 5 lines of random letters.” All participants were informed that they would then complete whichever they chose, and “after, we will ask you some questions about the task, including measures ensuring that you completed the task in full exactly as described.” Participants who chose the Gratitude Task were then given 5 min to write their message and then sent it to their friend. Participants who chose the Transcription Task were then given 5 min to exactly copy multiple lines of letters (e.g. “AJSDBSKLJFSDJJSSDSLDJSKLDJSKLSDJKLSJDKLSJDKLSJDSKLSDJLS”).

Upon completing their task, all participants rated their experienced happiness via six items, shown in random order and each rated from 1 (*disagree*) to 7 (*agree*): “This study put me in a good mood”; “This study made me feel happy”; “This study was a worthwhile use of time”; “This study was meaningful”; “I’m thankful to be in this study”; and “I enjoyed this study.”

### Experiment 3

We requested and yielded 1,000 participants from Prolific for $1.00. The experiment followed a two-single-factor design (Gap Length, within-subjects: Short, Long). All procedures were similar to experiment 2 aside from the design changes noted in the main text.

After making their choice, the experiment ended. Rather than collecting the experience measures in this experiment, here we more directly asked Long Gap participants about costs and benefits to their experience: Among Long Gap participants who chose the worse alternative, we asked them why they did not simply choose to text their friend instead (forced-choice: *because I’d prefer to wait for “the right time” to better mark the moment [even though the wait isn't enjoyable]* vs. *because I think the wait itself will be enjoyable [*e.g. *I’ll savor the anticipation]*).

At this point, we also asked participants to complete the individual difference measures for trait social anxiety (25) and trait rejection sensitivity (26), presented in random order.

### Experiment 4

We requested 500 “Cloud Approved” participants from Cloud Research for $0.50, yielding 501 participants. The experiment followed a two-single-factor design (Gap Length, between-subjects: Short, Long).

To begin, all participants imagined they “just did something you love to do,” something “that's true for you in your own life.” We then instructed them to imagine a certain amount of time has since passed, “simply because life got in the way”—for Short Gap participants “a short time”; for Long Gap participants, “a long time”—and that, “right here and now, there's suddenly a surprise chance to do it again.” All participants also reported what activity they brought to mind (open-ended text box), whether they had experienced such a gap before (forced-choice, randomly ordered: *yes* vs. *no*), and how long their gap was (forced-choice: *few days* vs. *few weeks* vs. *few months* vs. ∼*6* *months* vs. *∼1* *year* vs. *few years* vs. *∼decade* vs. *multiple decades*).

All participants then rated four items regarding what they would “prefer to do.” The first two were our key variables, randomly ordered: one about preferred delay (1 = *It’d be fine enough to do, doesn't feel dramatic*; 7 = *I’d prefer to delay this if I could, feels dramatic*) and one about demanded value (1 = *Doesn't need to be especially high value [*e.g. *especially enjoyable, memorable, meaningful, notable, etc.]*; 7 = *Needs to be especially high value [*e.g. *especially enjoyable, memorable, meaningful, notable, etc.]*). After, there were two other randomly ordered items: one about preference uncertainty (1 = *Doesn't need to involve me having clear self-insight into my likes and dislikes*; 7 = *Needs to involve me having clear self-insight into my likes and dislikes*) and one about rust (1 = *Doesn't need to involve me being extremely skilled and talented at performing it;* 7 = *Needs to involve me being extremely skilled and talented at performing it*).

### Experiment 5

We requested 600 prescreened-in “Cloud Approved” participants from Cloud Research for $1.00 (all of whom had at least one event to which they were still excitedly waiting to return; prescreen detailed below), yielding 599 participants. The experiment followed a three-single-factor design (Condition, between-subjects: Control, Present-Special, Past-Short).

We launched the experiment on Monday, 2021 April 19. To begin, we described “first-time back” experiences as in experiment 1. Then, we screened participants: We showed them a list of 10 randomly ordered events and asked whether they were still waiting to return to at least one of them (forced-choice, randomly ordered: *yes* vs. *no*): “Movies”; “Parties”; “Live sports”; “Live concerts”; “Local attractions”; “Dining”; “Bars”; “Travel”; “Shopping”; and “Pampering.”

Participants who indicated “*No*” were then screened out. Participants who indicated “*Yes*” then completed the experiment. As preregistered, we programmed the experiment such that it would keep running until we hit our requested number of 600 participants who all indicated “*Yes*.” This yielded a final recruitment of 641 participants, 599 of whom passed (93% retention).

All prescreened-in participants then saw the same list of events and read: “For this task, please focus on domains where you haven't yet had your “first-time back” experience. Which gap feels longest for you? (i.e. you’d rate this gap as feeling a full 10/10 very long).” Whatever they chose was piped into all prompts. Control participants then proceeded right to the dependent variable—the same one from experiment 1. Present-Special participants first read and wrote their response (open-ended text box) to the following prompt: “Spend a moment reflecting on the fact that any event like this can feel extra special, all things considered. In other words: It really doesn't matter what you fill it with, if you think about it. Who cares what you exactly do for it or how perfectly it goes. Precisely because it's been so long since you’ve last done it, your return will feel extra special either way! Right now, please reflect on this. Describe how your “first-time back” experience, no matter what, can feel extra special regardless of what you fill it with.” The prompt for Past-Short participants was: “Spend a moment reflecting on the fact that any event like this can feel not that long ago, all things considered. In other words: It really hasn't been that long, if you think about it. Who cares what the clock or calendar exactly says. Precisely because all our experiences are “recent” in the grand scheme of things, your return will feel not-that-long-ago either way! Right now, please reflect on this. Describe how your “first-time back” experience, no matter what, can feel not-that-long ago regardless of clock/calendar time.”

We also measured other variables simply for interest—the same ones from experiment 1 (e.g. feelings of safety; local status of reopenings). See Supplementary Information for details.

## Supplementary Material

pgaf156_Supplementary_Data

## Data Availability

All data, materials, and preregistrations are available at: https://osf.io/76czk/.

## Notes

a Of course, we did not randomly assign pandemic shutdowns nor the circumstances in which people could return from them, which forbids causal interpretation. We sacrificed this limitation for the high ecological validity afforded by this natural shock. All subsequent experiments entail randomly assigned manipulations, including randomly assigning people to different time gaps while holding constant all other stimulus features (e.g. experiment 4).

b We also checked the actual written gratitude letters and found no obvious differences across the gap conditions. For example, the length of the letters did not vary (across conditions, participants wrote about 100 words on average), nor did the length of time participants took to write them (across conditions, participants took about 5 min on average); see Supplementary Information for details and results. These findings help further rule out the “different kind of friend” alternative explanation (e.g. by suggesting that the gratitude task is similarly easy to do upon actually doing it, regardless of gap length). All original gratitude letters, as participants had written them, can be found in our data file.

c In total, 1,173 participants began the experiment, and our final sample of 1,000 were those who finished—suggesting that 85% of participants (1,000 of 1,173) could indeed summon identical friends in these ways. These findings help validate our prompts. They also further emphasize how common it indeed is for people to face long gaps of time between getting to do things they love to do, for no ill reason.
