# Supplementary material for: Lost time undermines return behavior
Source: PNAS Nexus. 2025 Jun 24;4(6):pgaf156. doi: 10.1093/pnasnexus/pgaf156 (PMC12199251; doi:10.1093/pnasnexus/pgaf156)
Supplement: pgaf156_Supplementary_Data [file pgaf156_supplementary_data.docx]

Supplementary Information for:

*Lost Time Undermines Return Behavior*

**EXPERIMENT 1**

1. Main results (full output): We conducted a Repeated-Measures Logistic Regression via SPSS GEE, entering participant as a subject variable, Event as a within-subjects variable, Gap Length (1-10) as a predictor variable, and Choice (sufficient-now vs. special-later) as the dependent variable. This produced an incidental main effect of Event, Wald = 97.99, df = 4, *p* < .001. Critically, there was also the hypothesized main effect of Gap Length, Wald = 33.73, df = 9, *p* < .001—which was *not* qualified by an interaction with Event, Wald = 41.25, df = 36, *p* = .252. We also conducted separate logistic regressions for each of the 5 events. Participants became more likely to choose special-later (vs. sufficient-now) as perceived gap length increased—for returning to restaurants (*B* = .17, SE = .04, *p* < .001); and to parties (*B* = .16, SE = .04, *p* < .001); and to travel (*B* = .14, SE = .04, *p* < .001); and to family (*B* = .10, SE = .03, *p* = .002). The effect for returning to movie theaters was not statistically significant (*B* = .02, SE = .05, *p* = .704).
2. Other variables: Recall that, for each event, we first asked participants to report whether they have already had their first-time back experience vs. whether they are still waiting:
   1. Most participants indicated “not yet”: Returning to Restaurants (55% not yet, 277 of 500 vs. 45% already, 223 of 500); Theaters (94% not yet, 468 of 500 vs. 6% already, 32 of 500); Parties (84% not yet, 421 of 500 vs. 16% already, 79 of 500); Travel (75% not yet, 377 of 500 vs. 25% already, 123 of 500); and Family (58% not yet, 290 of 500 vs. 42% already, 210 of 500).
   2. As reported in the main text, this variable does not interact with any result. Among “not yet”-responses only (*n* = 1,833 observations, of all 2,500; 73%), we find the same incidental effect of Event, Wald = 1080.94, df = 4, *p* < .001; the key effect of How Long, Wald = 691.09, df = 9, *p* < .001; and again no interaction, Wald = 33.37, df = 32, *p* = .400. Likewise, among “already”-responses only (*n* = 667 observations, of all 2,500; 27%), we find the same incidental effect of Event, Wald = 40.32, df = 4, *p* < .001; the key effect of How Long, Wald = 1222.84, df = 9, *p* < .001; and again no interaction, Wald = 33.55, df = 31, *p* = .345.
3. Other variables: “When did this particular experience happen for you?” (shown to “already had”-P’s) vs. “When do you think this particular experience will happen for you?” (for “not yet”-P’s), rated from 1 (*very close to now*) to 10 (*very far from now*):
   1. Restaurants: *M*_AlreadyHad_ = 4.20, *SD* = 2.62 vs. *M*_NotYet_ = 6.20, *SD* = 2.40
   2. Theaters: *M*_AlreadyHad_ = 5.13, *SD* = 2.42 vs. *M*_NotYet_ = 7.15, *SD* = 2.37
   3. Parties: *M*_AlreadyHad_ = 4.89, *SD* = 2.61 vs. *M*_NotYet_ = 6.93, *SD* = 2.31
   4. Travel: *M*_AlreadyHad_ = 4.45, *SD* = 2.71 vs. *M*_NotYet_ = 6.59, *SD* = 2.51
   5. Family: *M*_AlreadyHad_ = 4.47, *SD* = 2.87 vs. *M*_NotYet_ = 5.95, *SD* = 2.44
4. Other variables: “Thinking about the kinds of fun activities we asked you about in this study: In general, are these kinds of things around you opened back up? (i.e., regardless of your own personal feelings, etc.—are they objectively opened back up for people?)”
   1. 38% (192 of 500) said “Yes”
      1. 16% (31 of 192) specified, “Just now”
      2. 54% (103 of 192) specified, “For a little bit”
      3. 30% (58 of 192) specified, “For a while”
   2. 9% (44 of 500) said “No”
      1. 4% (2 of 44) specified, “Any moment now”
      2. 64% (28 of 44) specified, “Not for a little bit”
      3. 32% (14 of 44) specified, “Not for a while”
   3. 53% (264 of 500) said “Somewhere in between”
      1. 17% (46 of 264) specified, “Any moment now”
      2. 60% (157 of 264) specified, “Not for a little bit”
      3. 23% (61 of 264) specified, “Not for a while”
5. Other variables: “In general, do you personally feel safe/ready/etc. returning to these kinds of things? What is your own personal feeling on the matter?”
   1. 25% (123 of 500) said “Yes”
      1. 10% (12 of 123) specified, “Just now”
      2. 39% (48 of 123) specified, “For a little bit”
      3. 51% (63 of 123) specified, “For a while”
   2. 45% (227 of 500) said “No”
      1. 3% (7 of 227) specified, “Any moment now”
      2. 43% (98 of 227) specified, “Not for a little bit”
      3. 54% (122 of 227) specified, “Not for a while”
   3. 30% (150 of 500) said “Somewhere in between”
      1. 11% (17 of 150) specified, “Any moment now”
      2. 70% (105 of 150) specified, “Not for a little bit”
      3. 19% (28 of 150) specified, “Not for a while”
6. Other variables: All participants reported any technical difficulties or confusions (forced-choice: *no* vs. *yes [explain]*); completed an attention check regarding which event was not rated (forced-choice: *restaurants* vs. *theaters* vs. *parties* vs. *travel* vs. *family* vs. *celebrities*); completed an honesty check regarding whether we should trust their data (forced-choice: *yes* vs. *no*); and could share any thoughts via an open-ended text box.
   1. Technical difficulties/confusions: 99% (496 of 500) said “No”
   2. Attention check: 99% (493 of 500) passed
   3. Honesty check: 99% (498 of 500) passed
   4. Open-ended thoughts: All 500 responses are in the data file, verbatim as written.
7. Re-running our main analyses, but with all exclusions and controls:
   1. Key effect of “How Long” on Choice
      1. via Logistic Regression: Wald = 18.46, df = 9, *p* = .030.

**EXPERIMENT 2**

1. Further task details: Participants freely made their task choice. As faithfully described, participants who chose the Gratitude Task were then given 5 minutes (enforced via a visual countdown timer) to craft their message in an open-ended text box, and then submitted this message to us. We re-showed participants their message and instructed them to copy-and-paste it as is, and send it to their friend then and there (either via email, text, or social media). We again reminded them that “the following screens will include various checks to ensure you’ve indeed sent this note of gratitude. Do not click >> until you’ve actually sent it.” The >> button was disabled for a minimum of 15 seconds. After sending, they returned to our survey where they had to check off, “Ok, I just sent my gratitude note; I’m ready to proceed,” in order to continue to the rest of the experiment.

Also as faithfully described, participants who chose the Transcription Task were then given 5 minutes (enforced via a visual countdown timer) to copy a matrix of letters exactly as they were listed. All of these participants saw the same set of 5 target lines:

Line 1: AJSDBSKLJFSDJJSSDSLDJSKLDJSKLSDJKLSJDKLSJDKLSJDSKLSDJLS

Line 2: QPLLWQLPEULWRYEWTFLYHNBXNZBVASAJKLSDLLXSXSXSDHSUYDF

Line 3: TYATYERWTSQWMDNVCXCNXMNBZXZTXSMDSMTRQRQETWSMXSSM

Line 4: NNNNNNPSDUWMDSMDAKAAAAAATYQWQMMDMNDNDSPPPPSDEDX

Line 5: BCCCCVVVQWWRRTYPLAAEZZZJHGFTWBVBVBKJLDDSFDDFPTQNFG

Underneath were 5 text boxes corresponding to each of the 5 target lines, and participants had to copy each one exactly as presented. The target lines were shown as an image file, thus preventing participants from copying-and-pasting their responses. After completing this task and submitting their transcriptions, they continued to the rest of the experiment.

1. Other variables: All participants rated their closeness to the friend, from 1 (*at our peak, we weren’t very close*) to 7 (*at our peak, we were very close*); reported how long it had been since their last contact (open-ended text box); reported any technical difficulties or confusions (forced-choice: *no* vs. *yes [explain]*); completed an attention check regarding their study condition (forced-choice: *brought to mind someone I’ve not communicated with very recently* vs. *brought to mind someone I’ve not communicated with for a very long time*); completed an attention check for their chosen task (forced-choice: *Gratitude Task* vs. *Transcription Task*); and completed an honesty check regarding whether we should trust their data (forced-choice: *yes* vs. *no*). In addition, Long Gap participants reported what best explained the gap (forced-choice: *nothing “bad” per se; life simply got in the way* vs. *something “bad”; e.g., we fought/ended things on a sour note*).
   1. Closeness: *M*_ShortGap_ = 6.49 of 7, *SD* = .80 vs. *M*_LongGap_ = 6.32 of 7, *SD* = .90, *t*(198) = 1.37, *p* = .171.
   2. How long: Excluding non-specific responses (e.g., “A few months”), we recorded 175 responses (out of 200 total; 88% retainment), which we converted to seconds for analysis. Long Gap participants reported a longer gap (*M* = 40,948,146.46 sec, *SD* = 59,871,539.54; ~1 year) than Short Gap participants (*M* = 849,567.90 sec, *SD* = 4,832,491.52; ~1 week), *t*(173) = 6.12, *p* < .001.
   3. Technical difficulties/confusions: 98% (195 of 200) said “No”
   4. Attention check for condition: 95% (190 of 200) passed
   5. Attention check for chosen task: 99% (197 of 200) passed
   6. Honesty check: 93% (185 of 200) passed
   7. Long Gap best explained: Most chose “*nothing bad*” (93%, 95 of 102) rather than “s*omething bad*” (7%, 7 of 102). When re-running our analyses while excluding these 7 latter participants, all the patterns hold: Activity Choice, χ^2^ (1, *N* = 193) = 3.81, *p* = .051; Experienced Happiness, *t*(191) = 2.66, *p* = .009.
2. Other analyses (task differences): Perhaps merely bringing to mind a long gap in close contact reduces experienced happiness. To tease apart this contribution, we re-ran our main analysis (testing the effect of Gap condition on Experienced Well-Being) via Linear Regression, controlling for Task Choice as a covariate. In isolation, Gap significantly predicted Experienced Well-Being, *B* = .42, *SE* = .15, *p* = .006 (duplicating our main analysis)—yet this effect was cut in half when controlling for Task Choice, *B* = .20, *SE* = .11, *p* = .060. These results suggest it was Long Gap participants’ choice to avoid the Gratitude Task that was the primary spoiler of well-being, as hypothesized. Indeed, regardless of Gap condition, the Gratitude Task proved far better for well-being (among all 96 participants who chose it: *M* = 5.50, *SD* = 1.52) than the dull Transcription Task (among all 104 participants who chose it: *M* = 2.34, *SD* = 1.51), *t*(198) = 14.73, *p* < .001.
3. Other analyses (gratitude message differences): Among participants who chose the Gratitude Task, there were no differences between conditions in the overall length of their messages (*M*_ShortGap_ = 114.06 words, *SD* = 62.19 vs. *M*_LongGap_ = 94.71 words, *SD* = 59.53), *t*(94) = 1.54, *p* = .127; nor in how long it took for them to write their messages (*M*_ShortGap_ = 12.37 minutes, *SD* = 49.98 vs. *M*_LongGap_ = 5.46 minutes, *SD* = .69), *t*(94) = .90, *p* = .373; Short Gap becomes *M* = 5.57 minutes, *SD* = 1.23 after removing 1 outlier); nor in how long it took for them to send their messages (*M*_ShortGap_ = 40.57 seconds, *SD* = 41.14 vs. *M*_LongGap_ = 49.42 seconds, *SD* = 60.47), *t*(94) = .85, *p* = .397). All 96 messages are in the data file, verbatim as written.
4. Re-running our main analyses, but with all exclusions and controls:
   1. Experienced Happiness
      1. via Univariate GLM: *F*(1, 156) = 5.90, *p* = .016.
   2. Activity Choice
      1. via Logistic Regression: *B* = .36, SE = .17, *p* = .036.
5. Separate follow-up experiment: For additional confirmation that people can bring to mind a *close* friend, who they have *not* contacted for a long time, for *no* negative reasons—We recruited 100 unique undergraduates from the same institution via a separate experiment (71% women; 71% non-White; *M*_age_ = 21; 99% undergraduate students). They were given the same Long Gap prompt described above, i.e., to think of a friend who they feel close to and care about, and can easily reach via phone, email, or social media, and yet, for no particular reason, have not spoken with for a long time. They listed the initials of such a friend in their own life, and then rated this task and the notion of such a friend via 5 questions: These participants, who were recruited from the same population from our main experiment, indeed found it *easy* to bring to mind such a friend (*M* = 5.45, *SD* = 1.68; 1 = *difficult*, 7 = *easy*); they thought that, for people in general, it was *common* (*M* = 5.84, *SD* = 1.22; 1 = *rare*, 7 = *common*) and *normal* (*M* = 2.24, *SD* = 1.57; 1 = *normal*, 7 = *weird/unusual*) to have at least one such a friend; and thought that getting back in touch with such a friend would make *themselves* happy (*M* = 5.86, *SD* = 1.06; 1 = *not happy*, 7 = *happy*), and also *their friend* happy (*M* = 5.53, *SD* = 1.23; 1 = *not happy*, 7 = *happy*); as revealed via one-sample *t*-tests against each of these scale midpoints, all *t*s ≥ 8.60, *p*s ≤ .001). We also asked these participants to explain why they experienced their own long gap, despite no negative reason for it; common responses included geographical distance; busyness/scheduling conflicts; and different schools. Almost none of these participants (3%, 3 of 100) mentioned any negative reason (e.g., a sour ending or a changing taste). All original files for this follow-up experiment are on our OSF page.

**EXPERIMENT 3**

1. Further task details: To begin the experiments, all participants generated an example of both kinds of friend (a Short Gap friend and a Long Gap friend), which we asked them to do one at a time in random order. As we noted in the main text, our prompts explicitly instructed participants to bring to mind identical kinds of friends aside from the gap. Then, when participants made their activity choice, we described the alternative task (i.e., the non-friend option) as a “longer task, involving clicking through a boring website.” Upon completion of the experiment, we instructed those participants who chose the texting task to indeed text their friend as described, and those participants who chose alternative task to click through a website that they find boring.
2. Other variables: All participants reported how long it had been since their last text with each friend (open-ended text boxes); reported any technical difficulties or confusions (forced-choice: *no* vs. *yes [explain]*); completed an attention check regarding their study condition (forced-choice: *chose between boring task and texting short-gap friend* vs. *chose between boring task and texting long-gap friend*); completed an attention check for their chosen task (forced-choice: *boring task* vs. *friend task*); and completed an honesty check regarding whether we should trust their data (forced-choice: *yes* vs. *no*). In addition, Long Gap participants who chose not to text their friend reported why they chose this (forced-choice: *because I’d prefer to wait for “the right time” to better mark the moment [even though the wait isn’t enjoyable]* vs. *because I think the wait itself will be enjoyable [e.g., I’ll savor the anticipation]*). Finally, all participants completed the individual difference measures for trait social anxiety (*24*) and trait rejection sensitivity (*25*), presented in random order.
   1. How long: Excluding non-specific responses (e.g., “A few months”), we recorded 1,901 responses (out of 2,000 total; 95% retainment), which we converted to seconds for analysis. Participants reported they had a longer gap with Long Gap friends (*M* = 31,059,088.30 sec, *SD* = 48,928,380.20; ~1 year) than Short Gap friends (*M* = 664,106.31 sec, *SD* = 6,188,238.49; ~1 week), *t*(928) = 18.60, *p* < .001.
   2. Technical difficulties/confusions: 99% (990 of 1,000) said “No”
   3. Attention check for condition: 94% (944 of 1,000) passed
   4. Attention check for chosen task: 98% (980 of 1,000) passed
   5. Honesty check: 99% (994 of 1,000) passed
   6. Long Gap why avoid friend: Most chose “*wait unenjoyable*” (92%, 247 of 268) rather than “wait enjoyable” (8%, 21 of 268), χ^2^ (1, *N* = 268) = 190.58, *p* < .001.
3. Re-running our main analyses, but with all exclusions and controls:
   1. Activity Choice
      1. via Logistic Regression: *B* = .76, SE = .14, *p* < .001.

**EXPERIMENT 4**

1. Main results (full output): The full output for the other two items are as follows. Perceived uncertainty in one’s preferences: *M*_ShortGap_ = 3.66, *SD* = 1.67 vs. *M*_LongGap_ = 4.34, *SD* = 1.66), *t*(499) = 4.56, *p* < .001. Perceived rust in one’s abilities: *M*_ShortGap_ = 3.27, *SD* = 1.65 vs. *M*_LongGap_ = 3.78, *SD* = 1.76), *t*(499) = 3.33, *p* < .001.
2. Other variables: All participants reported any technical difficulties or confusions (forced-choice: *no* vs. *yes [explain]*); completed an attention check regarding what the study was about (forced-choice: *gaps in time between enjoyable events* vs. *how to write a college term paper* vs. *the history of rocks*); completed an attention check regarding their study condition (forced-choice: a *short time gap* vs. *a long time gap*); and completed an honesty check regarding whether we should trust their data (forced-choice: *yes* vs. *no*). We also asked all participants to describe what they brought to mind via an open-ended text box; to indicate whether they ever faced such a gap (forced-choice: *yes* vs. *no*); and to report their imagined gap length (forced-choice: forced-choice: *few days* vs. *few weeks* vs. *few months* vs. ~*6 months* vs. *~1 year* vs. *few years* vs. *~decade* vs. *multiple decades*).
   1. Technical difficulties/confusions: 99% (495 of 501) said “No”
   2. Attention check for study topic: 97% (488 of 501) passed
   3. Attention check for condition: 90% (452 of 501) passed
   4. Honesty check: 99% (496 of 501) passed
   5. Activity imagined: All 501 responses are in the data file, verbatim as written.
   6. Faced the gap: 89% (444 of 501) said “Yes”
   7. Length of gap, Short Gap condition: Few days (45%, 112 of 247); Few weeks (36%, 89 of 247); Few months (11%, 26 of 247); ~6 months (3%, 8 of 247); ~1 year (2%, 6 of 247); Few years (2%, 5 of 247); ~Decade (1%, 1 of 247); Multiple decades (0%, 0 of 247).
   8. Length of gap, Long Gap condition: Few days (2%, 4 of 254); Few weeks (7%, 17 of 254); Few months (18%, 46 of 254); ~6 months (17%, 44 of 254); ~1 year (13%, 33 of 254); Few years (32%, 81 of 254); ~Decade (8%, 21 of 254); Multiple decades (3%, 8 of 254).
3. Re-running our main analyses, but with all exclusions and controls:
   1. Preferred Delay
      1. Key effect of Gap Length: *F*(1, 386) = 15.28, *p* < .001
   2. Demanded Value
      1. Key effect of Gap Length: *F*(1, 386) = 54.23, *p* < .001
   3. Perceived Preference-Uncertainty
      1. Key effect of Gap Length: *F*(1, 386) = 27.05, *p* < .001
   4. Perceived Rust
      1. Key effect of Gap Length: *F*(1, 386) = 11.60, *p* < .001.
   5. Mediation via Demanded Value
      1. On its own: *B* = .67, SE = .11, 95% *CI*_Boot_ [.48, .89]
      2. Controlling the other two items: *B* = .21, SE = .06, 95% *CI*_Boot_ [.11, .33]

**EXPERIMENT 5**

1. Main results (full output): For the overall model, we conducted a Logistic Regression with Condition (3 levels) predicting Choice (2 levels), which yielded the hypothesized effect of Condition, Wald = 12.28, df = 2, *p* = .002. Moreover, in addition to the key pairwise comparisons reported in the main text, the final one (Present-Special vs. Past-Short) is as follows: *B* = .66, SE = .16, *p* = .002.
2. Further task details: To begin, all prescreened-in participants saw the same list of events and read: “For this task, please focus on domains where you haven’t yet had your ‘first-time back’ experience. Which gap feels longest for you? (i.e., you’d rate this gap as feeling a full 10/10 very long).” Whatever they chose was piped into all prompts. From most chosen to least chosen: Travel (chosen by 25% of participants, 152 of 599); Concerts (20%, 119 of 599); Movies (13%, 78 of 599); Restaurants (12%, 71 of 599); Sporting events (9%, 55 of 599); Local attractions (6%, 35 of 599); Parties (6%, 34 of 599); Bars (4%, 24 of 641); Pampering (3%, 18 of 599); and Shopping (2%, 13 of 599).

For both of the re-construal tasks, participants saw their given prompt and were then instructed to write a minimum of 200 characters (“~3-5 sentences”) in response, via an open-ended text box. The >> button was disabled for a minimum of 30 seconds.

1. Other variables: “Thinking about the kinds of fun activities we asked you about in this study: In general, are these kinds of things around you opened back up? (i.e., regardless of your own personal feelings, etc.—are they objectively opened back up for people?)”
   1. 32% (189 of 599) said “Yes”
      1. 15% (29 of 189) specified, “Just now”
      2. 62% (116 of 189) specified, “For a little bit”
      3. 23% (44 of 189) specified, “For a while”
   2. 15% (92 of 599) said “No”
      1. 14% (13 of 92) specified, “Any moment now”
      2. 51% (47 of 92) specified, “Not for a little bit”
      3. 35% (32 of 92) specified, “Not for a while”
   3. 53% (318 of 599) said “Somewhere in between”
      1. 20% (63 of 318) specified, “Any moment now”
      2. 60% (191 of 318) specified, “Not for a little bit”
      3. 20% (64 of 318) specified, “Not for a while”
2. Other variables: “In general, do you personally feel safe/ready/etc. returning to these kinds of things? What is your own personal feeling on the matter?”
   1. 34% (205 of 599) said “Yes”
      1. 27% (56 of 205) specified, “Just now”
      2. 41% (84 of 205) specified, “For a little bit”
      3. 32% (65 of 205) specified, “For a while”
   2. 37% (219 of 599) said “No”
      1. 6% (13 of 219) specified, “Any moment now”
      2. 43% (94 of 219) specified, “Not for a little bit”
      3. 51% (112 of 219) specified, “Not for a while”
   3. 29% (175 of 599) said “Somewhere in between”
      1. 9% (16 of 175) specified, “Any moment now”
      2. 69% (121 of 175) specified, “Not for a little bit”
      3. 22% (38 of 175) specified, “Not for a while”
3. Other variables: All participants reported any technical difficulties or confusions (forced-choice: *no* vs. *yes [explain]*); completed an attention check regarding what the study was about (forced-choice: *Returning to fun after the pandemic shutdown* vs. *YouTube* vs. *William James*); completed an attention check regarding whether they completed a writing task (forced-choice: *Yes* [and then, if chosen: “What about?”: *Past-Short* *prompt* vs. *Present-Special prompt*] vs. *No*); completed an honesty check regarding whether we should trust their data (forced-choice: *yes* vs. *no*); and could share any thoughts via an open-ended text box.
   1. Technical difficulties/confusions: 99% (591 of 599) said “No”
   2. Attention check for study topic: 99% (595 of 599) passed
   3. The complete attention check for the writing task: 99% (591 of 599) passed
   4. Honesty check: 99% (596 of 599) passed
   5. Open-ended thoughts: All 599 responses are in the data file, verbatim as written.
4. Other analyses (re-construal task differences): When comparing the two re-construal conditions, there were no differences in the overall length of participants’ written responses (*M*_PresentSpecial_ = 68.23 words, *SD* = 23.45 vs. *M*_PastShort_ = 70.65 words, *SD* = 26.09), *t*(372) = .94, *p* = .347; nor in how long it took for participants to write their responses (*M*_PresentSpecial_ = 8.49 minutes, *SD* = 4.53 vs. *M*_PastShort_ = 8.57 minutes, *SD* = 5.21), *t*(372) = .15, *p* = .879). All 374 responses are in the data file, verbatim as written.
5. Re-running our main analyses, but with all exclusions and controls:
   1. Key effect of Condition on Choice
      1. via Logistic Regression: Wald = 12.24, df = 2, *p* = .002
6. Separate follow-up experiment: To further confirm that our writing manipulations indeed worked as intended, we conducted a separate follow-up experiment with a manipulation check. Two days after the original experiment (also in April 2021), we recruited new participants from the same population (*N* = 330, yielding 301 screened participants: 54% women; 25% non-White; *M*_age_ = 38.91) as external raters. They followed the same procedures—assigned to Control, Present-Special, or Past-Short conditions—except the experiment stopped short of the dependent variable. Instead, at this point participants rated their experience on various dimensions, each from 1 (*definitely NOT feeling like this at the moment*) to 7 (*definitely AM feeling like this at the moment*). See our OSF page for all results; here we highlight two key insights. First, the two writing tasks indeed varied in their intended ways: The Present-Special prompt uniquely boosted participants’ perceptions that their “*first-time back experience would be special, no matter what*” (*M*_PresentSpecial_ = 5.70, *SD* = 1.44 vs. *M*_PastShort_ = 4.94, *SD* = 1.60 vs. *M*_Control_ = 5.44, *SD* = 1.47; *F*(2, 298) = 5.88, *p* = .003), while the Past-Short prompt uniquely boosted participants’ perceptions that “*the gap since last time hasn’t been that long*” (*M*_PastShort_ = 4.43, *SD* = 1.70 vs. *M*_PresentSpecial_ = 2.34, *SD* = 1.58 vs. *M*_Control_ = 2.38, *SD* = 1.79; *F*(2, 298) = 46.85, *p* < .001). These serve as basic manipulation checks. Second, the two writing tasks *did not* vary in unintended ways: There were no differences on items like how *difficult*, *believable*, *realistic, fun*, or *interesting* the task was (*p*s ≥ .103). Together, these findings suggest the different effects of the tasks on return behavior are not due to incidental reasons. All original files for this follow-up experiment are on our OSF page.
